# Characterization of the Bacteriophage-Derived Endolysins PlySs2 and PlySs9 with In Vitro Lytic Activity against Bovine Mastitis *Streptococcus uberis*

**DOI:** 10.3390/antibiotics9090621

**Published:** 2020-09-19

**Authors:** Niels Vander Elst, Sara B. Linden, Rob Lavigne, Evelyne Meyer, Yves Briers, Daniel C. Nelson

**Affiliations:** 1 Laboratory of Biochemistry, Department of Pharmacology, Toxicology and Biochemistry, Faculty of Veterinary Medicine, Ghent University, 9820 Merelbeke, Belgium; Evelyne.Meyer@ugent.be; 2 Laboratory of Antimicrobial Discovery, Institute for Bioscience and Biotechnology Research, University of Maryland, Rockville, MD 20850, USA; sara@ibbr.umd.edu; 3 Laboratory of Gene Technology, Department of Biosystems, KU Leuven, 3001 Heverlee, Belgium; rob.lavigne@kuleuven.be; 4 Laboratory of Applied Biotechnology, Department of Biotechnology, Ghent University, 9000 Ghent, Belgium; yves.briers@ugent.be

**Keywords:** PlySs2, PlySs9, bacteriophage-derived endolysins, bovine mastitis, dairy industry, intramammary antibiotics, alternative antimicrobials

## Abstract

Bovine mastitis, an infection of the cow’s mammary gland, is frequently caused by *Streptococcus uberis* and causes major economic losses in the dairy industry. The intramammary administration of antibiotics currently remains the predominant preventive and therapeutic measure. These antimicrobial compounds, of which some are considered critical in human health care, are frequently applied as dry therapy resulting in their consistent overuse. Therefore, the use of antibiotics in the dairy sector is being questioned. We here identified two endolysins, i.e., PlySs2 and PlySs9, respectively derived from *Streptococcus suis* serotype-2 and -9 prophages, with lytic activity against *S. uberis* in an in vitro setting. Both endolysins gave clear lysis zones in spot-on-plate assays and caused a reduction of the optical density in a turbidity reduction assay. In depth characterization identified PlySs9 as the more potent endolysin over PlySs2 with a lower MIC value and about one additional log of killing. PlySs2 and PlySs9 were challenged to a panel of subclinical and clinical *S. uberis* milk isolates and were both able to lyse all strains tested. Molecular dissection of these endolysins in catalytic and cell wall binding subdomains resulted in major loss of killing and binding activity, respectively. Taken together, we here propose PlySs2 and PlySs9 as candidate compounds to the current antimicrobial arsenal known against bovine mastitis-causing *S. uberis* as future add-on or replacement strategy to the currently used intramammary antibiotics.

## 1. Introduction

Bovine mastitis, an infection of the cow’s udder, is the most important economic disease affecting dairy cattle [1,2]. This infection is typically caused by a large variety of microorganisms and has either a clinical or subclinical outcome. Mastitis is frequently caused by the Gram-positive pathogen *Streptococcus uberis* [3,4,5]. Although good hygiene practices during the milking process diminish the incidence of streptococcal bovine mastitis, a full eradication proves impossible [6]. Additional preventive measures such as vaccination and probiotics have also been proposed [7,8]. Although these measures lower the infection rate, they do not result in elimination of the disease [9]. Therefore, antibiotics are still the main preventive and therapeutic tool for bovine mastitis, resulting in overuse mainly due to their application as dry therapy [10,11]. Several of these formulations contain quinolones, macrolides and cephalosporines of the third and fourth generation, which are considered critical in human health care by the World Health Organization [12]. Moreover, antibiotics can persist in milk and affect the dairy product or starter cultures of e.g., cheese and yoghurt fermentations. Therefore, the milk of treated animals has to be discarded, resulting in food waste and a negative impact on the environment [10]. Last but not least, the emerging risk of antibiotic resistance development in the dairy industry drives the search for alternative and superior solutions [3,13]. With the consumer’s demand moving towards more sustainable dairy products, the role of antibiotics as a preventive and therapeutic measure in this sector is increasingly questioned.

The use of bacteriophages (phages) and their lytic enzymes have been proposed as strategies that may supplement the current antibiotic arsenal [14,15,16,17,18]. Although phage therapy was initially considered promising, more recent insights revealed several remaining hurdles i.e., unpredictable pharmacokinetics and -dynamics, large molecular size compared to antibiotics, low tissue penetration, narrow bacterial specificity, the necessity to replicate in vivo, elevation of the somatic cell count in the cow’s milk and inactivation of bacteriophage by whey proteins [19,20,21]. However, phage-derived enzymes such as tail-spike enzymes (i.e., depolymerases) and endolysins bypass these disadvantages [22,23]. Endolysins, the most well-studied phage-derived peptidoglycan hydrolases, have been shown to successfully kill bacteria in vitro, in various animal models and in humans [15,22,24]. Local, topical therapy as well as single intravenous injections are either recently available on the pharmaceutical market or in full development, albeit only for human applications [25,26]. Hence, endolysins offer an interesting potential within the context of veterinary medicine [14,16,17,18]. More specifically, several endolysins have already been proposed to target Gram-positive pathogens associated with bovine mastitis [27,28,29,30,31]. In the current study, two endolysins, named PlySs2 and PlySs9 as derivatives from prophages in the genomes of the porcine pathogen *Streptococcus suis*, were characterized as potential antimicrobial compounds against bovine mastitis-causing *S. uberis*. PlySs2 is known to display broad lytic activity against several streptococci i.e., *Streptococcus equi*, *Streptococcus agalactiae, Streptococcus dysgalactiae*, *Streptococcus pyogenes*, *Streptococcus sanguinis*, *Streptococcus pneumoniae* and group E streptococci [32,33,34]. In addition, its lytic activity was confirmed in an animal model showing eradication of *S. suis* from murine nasal mucosa with a > 4 log reduction [35]. In contrast, our group is the first to report PlySs9, but its protein sequence is 96.5% identical to PlySK1249, an endolysin derived from a *S. dysgalactiae* prophage [36]. The latter endolysin was able to lyse *S. dysgalactiae* and *S. agalactiae* in vitro and its efficacy and safety were already preclinically evaluated in vivo. Regardless, neither PlySs2 nor PlySs9 have been investigated in depth against bovine mastitis-causing *S. uberis*. Therefore, we evaluated the in vitro lytic activity of PlySs2 and PlySs9, as well as their individual subdomains, as potential therapeutics in the future treatment of bovine mastitis caused by *S. uberis*.

## 2. Results

### 2.1. Properties of PlySs2, PlySs9 and Their Individual Subdomains

**Figure 1:**
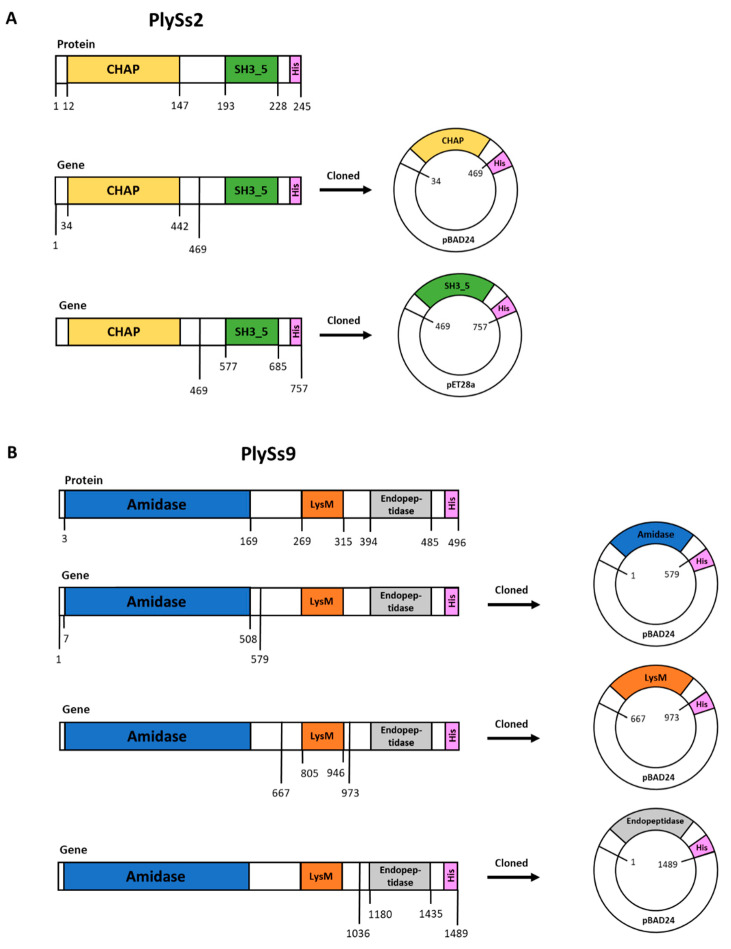
Schematic representation of the parental PlySs2 (**A**) and PlySs9 (**B**) protein sequences and their respective synthesized genes. PlySs2 consists of an N-terminal histidine-dependent amino hydrolase/peptidase (CHAP) and a C-terminal SH3_5 cell binding domain (CBD). PlySs9 has a LysM-based CBD and two catalytic domains; an N-terminal N-acetylmuramoyl-L-alanine amidase and a C-terminal endopeptidase. Subdomain boundaries regarding cloning into a pBAD24 or pET28a vector were selected to correspond to the linker regions such that predicted α-helices and β-sheets were not disrupted. To aid in downstream purification, the coding sequence for a polyhistidine tag was incorporated into the reverse primers used to amplify subdomain sequences (see also Table 2).

PlySs2 is 26.9 kDa in size and has an isoelectric point (pI) of 9.06. It consists of an N-terminal histidine-dependent amino hydrolase/peptidase (CHAP) catalytic domain and a C-terminal SH3_5 cell-wall binding domain (CBD). The CHAP and SH3_5 subdomains range from position 12–147 and 193–228, respectively, within the PlySs2 amino acid sequence (Figure 1A). PlySs9 is 54.1 kDa in size and has a pI of 9.08. It consists of one LysM-based CBD and two catalytic domains, an N-terminal N-acetylmuramoyl-L-alanine amidase and a C-terminal endopeptidase. The amidase subdomain ranges from position 3–169, the CBD from position 269–315 and the endopeptidase from position 394–485 (Figure 1B). 

**Figure A1:**
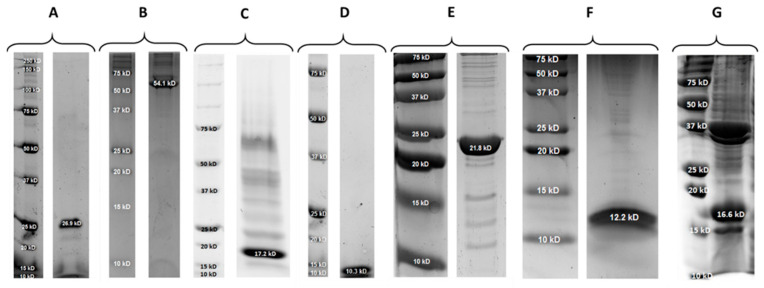
Confirmation by SDS-PAGE of the sizes (Left: protein ladder) and purity (Right: purified protein, the reported numbers represent the in silico calculated masses) of the constructs expressed and purified: PlySs2 (**A**), PlySs9 (**B**), PlySs2 CHAP (**C**), PlySs2 SH3_5 (**D**), PlySs9 amidase (**E**) and PlySs9 LysM (**F**). As PlySs9 endopeptidase (**G**) was present in the insoluble fraction, it was not purified. Gels consisted of 12.5% acrylamide and were stained with Coomassie brilliant blue.

The mass, pI’s and expression yields of full-length and subdomains for both PlySs2 and PlySs9 are represented in Table 1. The PlySs9 endopeptidase subdomain was not expressed in a soluble form and therefore its further purification and downstream assays could not be performed. The size and purity of all proteins were verified by SDS-PAGE (Figure A1).

### 2.2. Qualitative Assessment of the Lytic Activity of PlySs2, PlySs9 and Their Catalytic Subdomains

**Figure 2:**
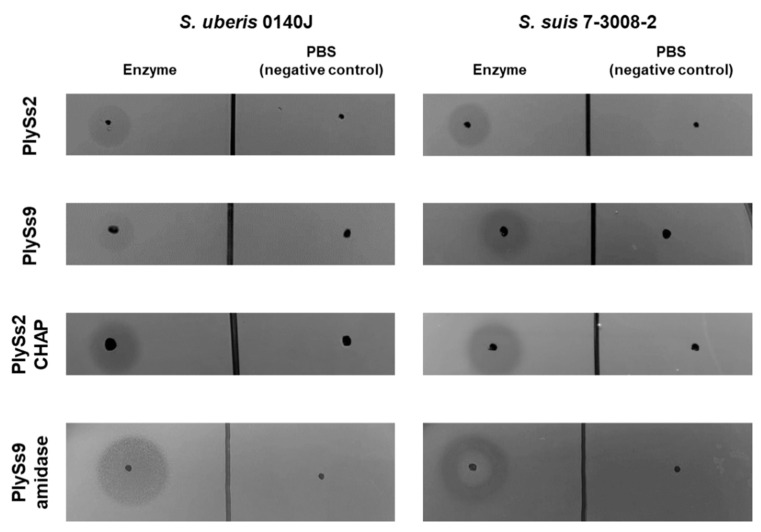
Qualitative spot-on-plate assays of PlySs2, PlySs9, PlySs2 CHAP and PlySs9 amidase on *S. uberis* 0140J and *S. suis* 7-3008-2 after incubation during 18 h at 37 °C. *S. suis* was included as positive control, phosphate buffered saline (PBS) served as negative control.

To confirm that expression and purification of both parental endolysins and their catalytic subdomains resulted in properly folded and active proteins, a spot-on-plate assay was performed with *S. uberis* as well as *S. suis*. The latter pathogen was included as a positive control, since it is the host of the bacteriophages from which the parental endolysins were derived. PBS buffer without protein was used as negative control. PlySs2, PlySs9, PlySs2 CHAP and PlySs9 amidase all gave clear lysis zones on both *S. uberis* and the positive control, while no lysis was observed in the negative control (Figure 2). Taken together, the results indicate full-length enzymes and catalytic subdomains were properly folded.

### 2.3. Quantitative Assessment of the Lytic Capacity of PlySs2, PlySs9 and Their Catalytic Subdomains

**Figure 3:**
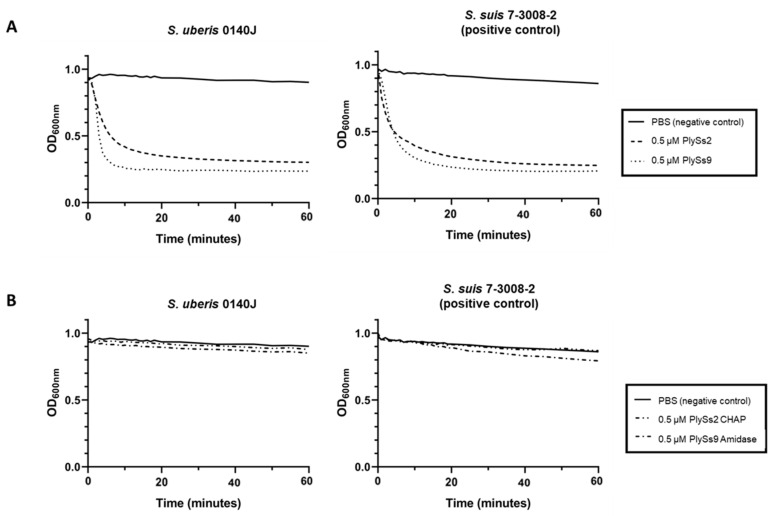
Turbidity reduction assays with 0.5 µM of PlySs2 and PlySs9 (**A**), and PlySs2 CHAP and PlySs9 amidase (**B**) on stationary phase *S. uberis* 0140J and *S. suis* 7-3008-2, the latter pathogen being included as a positive control. Bacteria in PBS served as negative controls.

**Figure 4:**
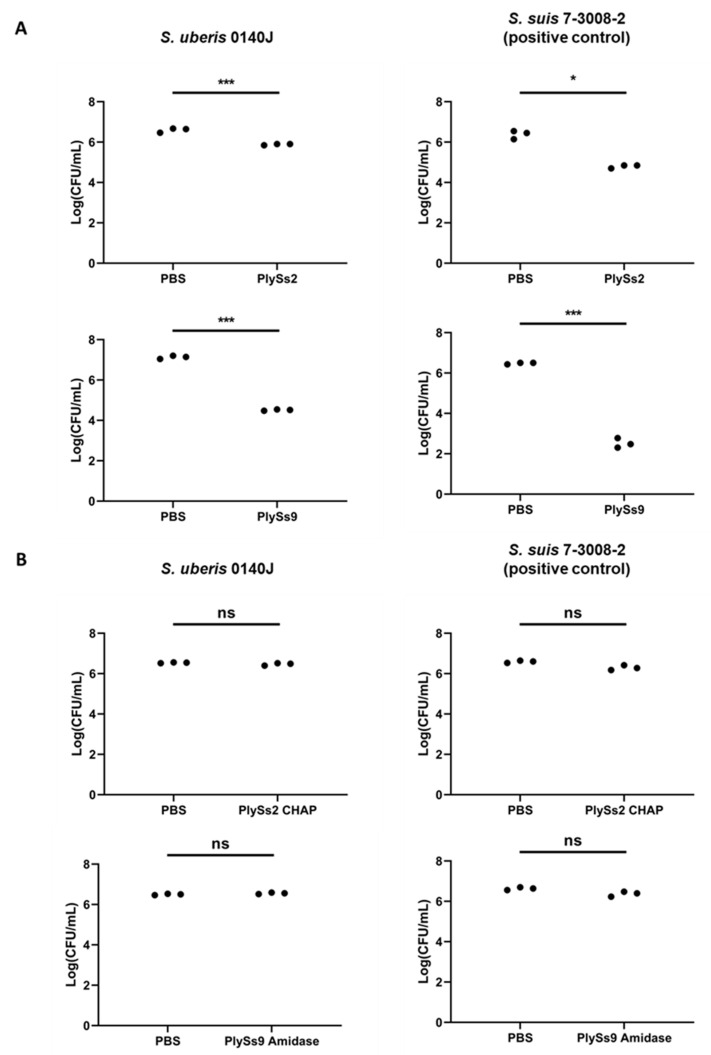
Time killing assays with PlySs2 and PlySs9 (**A**), and PlySs2 CHAP and PlySs9 amidase (**B**) for 2 h at 37 °C on stationary phase *S. uberis* 0140J. *S. suis* 7-3008-2 served as the positive control and bacteria in PBS were included as negative controls. 10^6^ CFU/mL were challenged with 0.5 µM enzyme. * indicates *p* < 0.05, *** indicates *p* < 0.001 and ‘ns’ indicates non-significant corresponding to a *p* > 0.05.

To quantify the lytic capacity of the parental endolysins and their catalytic subdomains, turbidity reduction assays (TRAs) and time killing assays (TKAs) were performed on *S. uberis* (Figure 3 and Figure 4, respectively). *S. suis* again served as the positive control, whereas bacteria in PBS again served as negative controls. For the TRAs, at equimolar concentrations of 0.5 µM (corresponding to 13.45, 27.05, 8.60 and 10.90 µg/mL for PlySs2, PlySs9, PlySs2 CHAP and PlySs9 amidase, respectively), PlySs2 and PlySs9 caused a decrease in OD_600_ (optical density measured at 600 nm) for stationary phase *S. uberis* that was similar to the positive control (Figure 3A). The negative controls did not show a notable decrease in OD_600_. In contrast, activity was low to negligible for the PlySs2 CHAP and PlySs9 amidase in this assay. The individual catalytic domains did not induce a drop in OD_600_ in the positive control either (Figure 3B), although a limited linear decrease in OD_600_ for the PlySs9 amidase was observed. Additionally, the observed enzymatic activity of both parental endolysins against *S. uberis* was quantified as described [37] and estimated 0.136 (ΔOD_600_/min)/µM with R^2^ = 0.965 and 0.176 (ΔOD_600_/min)/µM with R^2^ = 0.983 for PlySs2 and PlySs9, respectively.

Additional TKAs were performed to further evaluate the lytic capacity of both PlySs2 and PlySs9 at the same equimolar concentrations as described for the TRAs (Figure 4). PlySs2 lysed 1.09 ± 0.37 log of *S. uberis*, PlySs9 lysed 2.54 ± 0.08 log of the same strain (Figure 4A). Comparable or higher lytic capacities were observed for the positive control, i.e., 1.36 ± 0.25 log for PlySs2 and 3.86 ± 0.06 log for PlySs9, respectively (Figure 4A). Again, no significant decrease was observed for either PlySs2 CHAP or PlySs9 amidase (Figure 4B). In addition, no reduction in bacterial number for these individual catalytic domains was observed in the positive control (Figure 4B).

As a third quantitative parameter, the minimal inhibitory concentration (MIC) was determined for both the parental endolysins and their catalytic domains. The MIC values exceeded 5 µM (corresponding to 86, 109 and 134.5 µg/mL for PlySs2 CHAP, PlySs9 amidase and PlySs2, respectively), while the PlySs9 MIC was significantly lower i.e., 0.48 ± 0.16 µM (corresponding to 26.17 ± 8.80 µg/mL). Thus, only PlySs9 yielded an observable MIC under the concentrations tested.

### 2.4. Evaluation of the Pathogen-Binding Capacity of the PlySs2 and PlySs9 Cell-Wall Binding Domains

**Figure 5:**
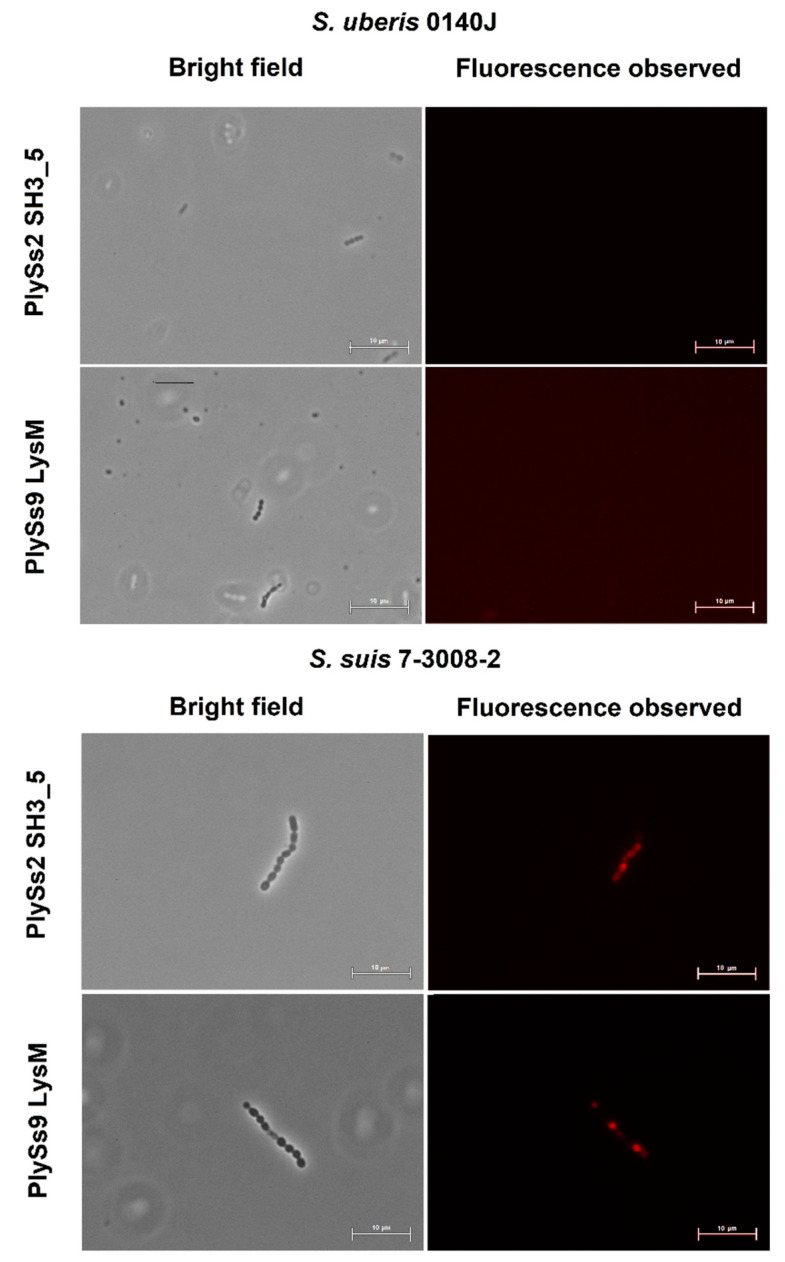
Bright field and fluorescent images of mid-log phase *S. uberis* 0140J observed by microscopy after incubation with the Alexa Fluor 555 conjugated PlySs2 SH3_5 and PlySs9 LysM cell wall binding subdomains. *S. suis* 7-3008-2 was included as positive control. Scale bars measure 10 µm.

After Alexa Fluor 555 conjugation to the CBDs of PlySs2 and PlySs9, (e.g., PlySs2 SH3_5 and PlySs9 LysM, respectively), no binding to *S. uberis* was observed (Figure 5). In contrast, the CBDs did bind to the *S. suis* positive control (Figure 5). As a confirmation test, two other *S. uberis* pathogens i.e., ATCC 27,958 and ATCC 700,407 were additionally evaluated and again no binding was observed.

### 2.5. Evaluation of the Lytic Activity of PlySs2 and PlySs9 against A Panel of Isolated (Sub)Clinical S. uberis Strains

**Figure 6:**
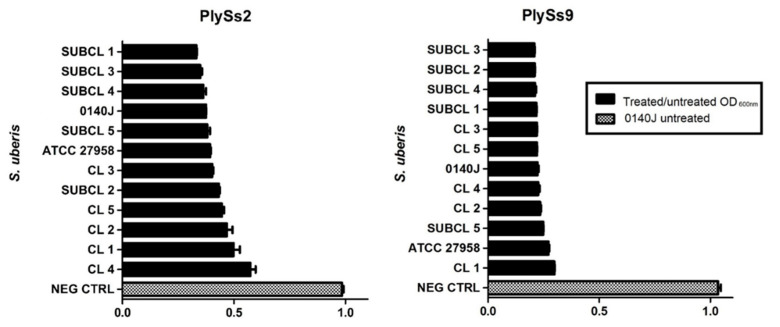
Reduction of the OD_600_ of multiple subclinical (SUBCL) and clinical (CL) *S. uberis* strains and the references *S. uberis* 0140J and ATCC 27,958 after 1 h incubation at 37 °C with 0.5 µM of PlySs2 and PlySs9. *S. uberis* 0140J in PBS served as the negative control (NEG CTRL).

To extrapolate the activity of PlySs2 and PlySs9 from the *S. uberis* 0140J reference strain to the same pathogen but currently occurring in infected dairy cattle, a panel of recently isolated subclinical and clinical *S. uberis* was composed and challenged by TRA with both endolysins at 0.5 µM during 1 h. The strains originated from various dairy farms and were freshly isolated from milk samples by the Milk Control Center of Flanders (Belgium). They were complementary verified by MALDI-TOF (data not shown) [38]. The reference strain *S. uberis* ATCC 27,985 was additionally included. All strains challenged were susceptible to both PlySs2 and PlySs9 as demonstrated by a reduction in the OD_600_ (Figure 6). *S. uberis* 0140J incubated in PBS served as the negative control. The observed reduction in OD_600_ was again the highest for PlySs9 on all strains challenged (Figure 6).

## 3. Discussion

Bovine mastitis is the most common production disease in the dairy industry and is frequently caused by the udder pathogen *S. uberis* [13]. Several preventive, prophylactic and therapeutic measures have been proposed to combat this infectious disease, but antibiotics currently remain the standard antimicrobial preventive and therapeutic treatment [6,9]. This systematic (over)use of antibiotics in the dairy sector is being questioned and as such, endolysins have been proposed as either replacement or add-on strategy [14,16,18]. We here characterized two endolysins, i.e., PlySs2 and PlySs9, with muralytic activity against bovine mastitis-causing *S. uberis*. Both endolysins showed activity against all strains of a panel consisting of clinical and subclinical bovine *S. uberis* isolates. In-depth characterization identified PlySs9 over PlySs2 as the more potent endolysin towards *S. uberis*, showing the lowest MIC value and an additional log of microbial killing capacity.

The spot-on-plate assay, TRA, TKA and determination of the MIC are four commonly used methods to demonstrate antimicrobial activity of endolysins [31,33,35,39]. However, these assays should be regarded complementary and are not directly comparable to each other [39]. A TKA is a direct measure of bacterial cell death, whereas a TRA is an indirect assay measuring a loss of turbidity in a bacterial solution, which can be inferred as corresponding to bacterial death [37]. However, the loss of turbidity relies on the light scattering phenomenon, which itself can be affected by osmotic pressure within the bacterial cell, buffer components and contributions of proteins, nucleic acids, cell wall fragments and capsule [37]. Concerning determination of the MIC, it should be noted that endolysins are enzymes characterized by a half-life and limited turn-over. When bacteria are challenged upon determination of the MIC, the enzyme can already be used or inactivated before all bacteria are killed. Consequently, the remaining bacteria will grow and a MIC cannot be observed. As such, endolysins can show very promising results in a spot-on-plate assay, TRA or TKA, but no MIC is observed. In our results, this was present for the full-length PlySs2 endolysin. Out of the complementary assays discussed, the spot-on-plate assay is by far the most sensitive, because the endolysin is undiluted and allowed to act on the bacterial target for 18 h. These bacteria are embedded in a nutrient-lacking agar and can therefore not grow. As a result, enzymes that often show little or no activity if challenged by TRA, TKA or MIC will show activity in the spot-on-plate assay given the higher sensitivity of this method. The presence of a clearing zone in the spot-on-plate assay combined with the fact that the enzyme was expressed as a soluble protein, is proof of proper folding. Therefore, the observation that the PlySs2 CHAP and PlySs9 amidase showed a lytic zone in the spot-on-plate assay, but deficient activity in the TRA, TKA and MIC assay, is not a contradictory finding but rather additionally underscores the importance of the CBD in binding the bacterial surface for these catalytic domains. However, it should also be noticed that the spot-on-plate assay has its limits. Diffusional differences in agarose are different for each endolysin and the size of the clearing zone is highly dependent on the molecular weight. Therefore, we here regarded the spot-on-plate assay a qualitative assay, rather than a quantitative method.

Molecular dissection of PlySs2 and PlySs9 into their separate catalytic domains and CBDs identified the catalytic subdomains with deficient lytic activity towards *S. uberis* compared to their parental counterparts. This finding corroborates the general observation on the activity of individual endolysin subdomains, i.e., catalytic subdomains often lack activity without their corresponding CBDs [22,33]. Nevertheless, notable exceptions to this general exist such as the endopeptidase domains of the phage endolysins LysK and PlyGBS [40,41]. These latter mentioned catalytic subdomains not only possess activity, but even have improved activity compared to their respective full-length endolysin. In this context, it can be noted that a limited linear reduction in OD_600_ was observed for PlySs9 amidase in the *S. suis* positive control. Nevertheless, our data suggest proper folding of these individual catalytic subdomains. As already discussed, lysis was observed in an agar-based assay after close contact for 18 h, whereas the other assays performed in solution for only 1–2 h did not reveal any significant activity. Our data therefore corroborate an earlier report that shows the importance of the corresponding CBD for full lytic activity of PlySs2 [33]. Comparing this deficient muralytic activity of the catalytic subdomains with that of the parental endolysins, our data overall suggest that the presence of CBDs is required for demonstration of full muralytic activity of PlySs2 CHAP and PlySs9 amidase. Still, no binding of the Alexa Fluor-conjugated CBDs was observed for *S. uberis*, although both CBDs did bind the *S. suis* positive control. Our findings for *S. uberis* resemble similar to studies with PlySs2 and *S. aureus*, in which the PlySs2 SH3_5 failed to bind the staphylococcal surface, yet the full-length enzyme retained high lytic activity toward *S. aureus* [33]. We suggest that the presence of the catalytic subdomains most likely is a necessity for optimal binding to the bacterial cell wall of *S. uberis*.

Notwithstanding the observed deficient activity of the individual subdomains, all subdomains had improved expression and purification yields compared to their respective parental counterparts, with the exception of PlySs9 endopeptidase that was expressed insolubly. Moreover, these individual subdomains were properly folded independently as was observed by spot-on-plate for the catalytic subdomains and by binding to *S. suis* for the CBDs. Therefore, these subdomains are amenable to engineering approaches to create chimeric constructs that may have improved characteristics compared to their wild type full-length endolysins. Such engineering efforts can be facilitated by recently introduced high-throughput assembly methods for chimeric endolysins [42].

In conclusion, PlySs2 and PlySs9 both have in vitro muralytic activity against a range of clinical and subclinical bovine mastitis *S. uberis* strains. In depth characterization further substantiated PlySs9 as the more potent endolysin, killing one additional log and showing a lower MIC than PlySs2. Additional molecular dissection of both endolysins showed that the individual subdomains, i.e., PlySs9 amidase, PlySs9 LysM, PlySs2 CHAP and PlySs2 SH3_5, have deficient killing and binding activity compared to their respective parental endolysin. Taken together, our data warrant the exploration of PlySs2 and PlySs9 as well as future engineering of their subdomains as candidate novel antimicrobial compounds against bovine Gram-positive mastitis-causing *S. uberis*.

## 4. Materials and Methods

### 4.1. Bacterial Strains and Culture Conditions

*S. uberis* ATCC BAA-854, also known as *S. uberis* 0140J, and *S. suis* 7-3008-2 (clinical isolate) were used as reference strains. Two other *S. uberis* strains, i.e., ATCC 27,958 and ATCC 700,407, were additionally included. All *S. uberis* strains used are bovine isolates. Streptococci were grown at 37 °C and 5% CO_2_ in Todd-Hewitt broth with the addition of 1% yeast extract (THY). Initial plasmid transformation was performed with *E. coli* DH5α (NEB) and protein expression was executed with *E. coli* BL21 (DE3) (NEB). *E. coli* were grown in Lysogeny Broth (LB) containing either 100.0 µg/mL carbenicillin or 50.0 µg/mL kanamycin. Selected plates that were used consisted of the same recipe, but 15.0 g/L agar was added.

### 4.2. Structural and Functional Bioinformatics of PlySs2 and PlySs9

PlySs2 and PlySs9 are putative peptidoglycan hydrolases, derived from prophage elements discovered in *S. suis*, that belong to serotypes 2 and 9, respectively [32,43]. The endolysin sequences discovered correspond to the streptococcal bacteriophages phi891591 and phiD12, respectively, with NCBI GenBank accession numbers AGF87539.1 and AGF87618.1. Analysis of the individual subdomains, secondary protein structure (α-helices and β-sheets) and three-dimensional structure were all predicted using Phyre2 [44]. The predicted molecular size and pI reported in Table 1 was calculated by the pI/Mw tool at ExPASy (https://web.expasy.org/compute_pi).

### 4.3. Plasmid Construction, DNA Manipulation and Cloning

The PlySs2 and PlySs9 coding sequences were chemically synthesized and codon optimized for expression in *E. coli* by ThermoFisher Scientific (Invitrogen Gene Art, Waltham, MA, USA) and a C-terminal polyhistidine (His_6_) tag was included for purification purposes. Cloning of the individual subdomains from the parental endolysins respected the architecture predicted by cleaving in the linker sequences, not disrupting any predicted α-helices or β-sheets. If needed, a start codon (ATG) or His_6_ tag was added to the construct by tail PCR (Table 2 and Figure 1). All constructs were initially cloned into the pBAD24 vector. The PlySs2 SH3_5 was subcloned into a gateway compatible pET28a vector using *EcoRI* and *SalI* restriction sites. Successful cloning was verified by Sanger sequencing the constructs (Psomagen, Rockville, MD, USA). The ApE program (University of Utah, Salt Lake City, UT, USA) was used for DNA sequence analysis and manipulations.

### 4.4. Expression Conditions and Protein Purification

Transformed *E. coli* BL21 (DE3) were grown as separate 1.5 L cultures in baffled Erlenmeyer flasks to an OD_600_ of 0.8–1.0. These cultures were induced with 0.25% L-arabinose (pBAD24) or 1 mM isopropyl β-D-1-thiogalactopyranoside (IPTG, pET28a) and shaken overnight (18 °C, 150 rpm). Next, bacteria were pelleted at 3300 g and resuspended in PBS with 10 mM imidazole and 1 mM phenylmethylsulfonyl fluoride (PMSF). Cells were sonicated on ice during 15 min (min). Insoluble cellular debris was removed by centrifugation in a JA-14 fixed angle rotor (Beckman Coulter, Brea, CA, USA) for 45 min at 25,000 g and 4 °C. Ni - NTA chromatography allowed purification of the protein from the centrifuged supernatant. Proteins were eluted from the nickel resin with PBS containing increasing concentrations of imidazole (20 mM, 50 mM, 100 mM, 250 mM and 500 mM). Imidazole-eluted fractions that contained the recombinant protein, as evaluated by SDS-PAGE, were combined and dialyzed for 4 h at 4 °C against 5.0 L PBS. All soluble proteins were stable in PBS (pH 7.4), except for the catalytic subdomain (i.e., CHAP domain, see results) of PlySs2 which was eluted from the nickel column with 500 mM imidazole in MES-buffer (125 mM MES, pH 6.1) and dialyzed against 3.0 L MES-buffer without imidazole (250 mM MES, pH 6.1). The cell-wall binding subdomains of both PlySs2 and PlySs9 needed an additional size exclusion purification step using an S-200 gel filtration column (Äkta, Marlborough, MA, USA).

### 4.5. Spot-on-Plate Assays, Turbidity Reduction Assays, Time Killing Assays and Minimal Inhibitory Concentrations

All assays used purified endolysins or endolysin subdomains. Spot-on-plate assays were performed by washing an overnight culture (18 h) of *S. uberis* or *S. suis* with PBS, followed by dissolving the bacterial pellet in 10 mL of 0.7% sterile liquid agarose and pouring it in petri dishes. Next, purified endolysins or PBS (negative control) were spotted on top of the agar and incubated for 18 h at 37 °C. For PlySs2 and PlySs9, 10 µL of a 1 µM enzyme in dialysis buffer was spotted on top of the agar, while this was 10 µL of a ≥15 µM solution for the catalytic subdomains. TRAs were performed by combining 100 µL of *S. uberis* or *S. suis* cells with an equal volume of 1 µM purified enzyme in PBS. Overnight grown cells were washed with PBS and resuspended with a 1:1 mixture with enzyme to an OD_600_ = 1. Next, the OD_600_ was measured every 15 s at 37 °C for 1 h, shaking the 96-well plate between each measurement. TKAs were performed by diluting an overnight culture, after standardization of the growth conditions, to 10^6^ colony forming units (CFU)/mL in PBS. A volume of 100 µL of diluted cells was combined with an equal volume of 1 µM purified enzyme and incubated for 2 h at 37 °C. In a next step, cells were serially diluted and spotted on THY plates. After overnight incubation, CFUs were counted. TRAs and TKAs were performed in triplicate in two independent experiments. Minimal inhibitory concentrations (MICs) were determined by diluting an overnight culture in double concentrated THY to 10^3^ CFU/mL. A 100 µL aliquot of the latter was then combined with an equal volume of aliquots containing different concentrations of the enzyme in a sterile 96-well plate for an 18 h incubation at 37 °C. MIC values were reported in µM to overcome the discrepancy in size of endolysins relative to antibiotics. MIC determination was performed in three independent experiments.

### 4.6. Alexa Fluor 555 Labeling and Fluorescence Microscopy

Alexa Fluor 555 labeling was performed according to the manufacturer’s protocol (Alexa Fluor 555 Protein Labelling Kit, ThermoFisher Scientific, Waltham, MA, USA). A 1 M sodium bicarbonate (pH 8.3) solution was used to reach the desired pH range (7.5–8.5) of the protein solution for optimal conjugation. Following activation using DMSO, Alexa Fluor 555 was added to the protein solution (2.5 mg/mL) and incubated for 1 h on a shaker (60–80 rpm) at room temperature. During this incubation, a disposable PD10 desalting column (GE Healthcare, Chicago, IL, USA) was loaded with resin and equilibrated with elution buffer. Next, conjugated protein was added to the column and eluted. Fractions that contained the conjugated protein were collected in 1.5 mL Eppendorf tubes. Subsequently, bacterial cells in mid-log phase were washed, resuspended in PBS and combined with an equal volume of Alexa Fluor 555 conjugated PlySs2 SH3_5 or PlySs9 LysM domains. Following 10 min of incubation at room temperature, cells were again washed with PBS and visualized by fluorescence microscopy (Eclipse 80i, Nikon, Melville, NY, USA). The fluorescence observed was measured at 620 nm as emission wavelength (540 nm as excitation wavelength).

### 4.7. Statistical Analysis

Unpaired *t*-tests were performed using Prism (GraphPad, San Diego, CA, USA) to calculate *p*-values. If *p* < 0.05, the observed difference was considered statistically significant.

## Figures and Tables

**Table 1:** Molecular size (kDa) and iso-electric point (pI) was calculated from the amino acid composition for each protein (see section material and methods) of the different proteins expressed and purified. The expression yield of the PlySs9 endopeptidase subdomain could not be determined, because it was expressed insolubly.

Protein	Molecular Size (kDa)	pI	Expression Yield (mg/L)
PlySs2	26.9	9.06	0.25
PlySs2 CHAP	17.2	7.10	2.60
PlySs2 SH3_5	10.3	9.57	1.10
PlySs9	54.1	9.08	0.12
PlySs9 Amidase	21.8	6.75	20.0
PlySs9 LysM	12.2	9.84	1.10
PlySs9 Endopeptidase	16.6	6.42	Not determined

**Table 2:** Forward and reverse primer sequences and their respective melting temperature (Tm) used to clone the individual subdomains from the parental endolysins by PCR. The coding sequence of the polyhistidine tag is underlined.

Subdomain Amplified by PCR	Forward Primer	Reverse Primer	Tm (°C)
PlySs2 CHAP	GAATTCATTATGGCA CAGGTTGGTAGCGGT	TCTAGATTAATGATGATGATGAT GATGCTGACGATAGCTTGCTGC	72
PlySs2 SH3_5	GAATTCATTATGAGC CGTAGCTATCGTGAA	TCTAGATTAATGATGATGATGAT GATGTTTAAAGGTGCCCCATGC	69
PlySs9 Amidase	GAATTCATTATGGGC AAACATCTGGTGATT	TCTAGATTAATGATGATGAT GATGATGCTCGGGGTGCCGCTG	68
PlySs9 LysM	GAATTCATTATGAAA GGTCGCACCTACAAA	TCTAGATTAATGATGATGATGAT GATGGGTCGGAATTGCGGTGGT	68
PlySs9 Endopeptidase	GAATTCATTATGAGC AATCCGTATGGTGGT	GATTTAATCTGTATCAGG	51
